# Robust 3D Eccentric Field Synthesis for OTA Testing via an Enhanced Spherical Vector Wave Approach

**DOI:** 10.3390/s26134012

**Published:** 2026-06-24

**Authors:** Jianchuan Wei, Zhanying Peng, Xiaoming Chen

**Affiliations:** School of Information and Communications Engineering, Xi’an Jiaotong University, Xi’an 710049, China; jianchuan.wei@stu.xjtu.edu.cn (J.W.); pzyrrr@stu.xjtu.edu.cn (Z.P.)

**Keywords:** over-the-air (OTA) testing, multiprobe anechoic chamber (MPAC), eccentric testing, three-dimensional (3D) field synthesis

## Abstract

Traditional over-the-air (OTA) testing typically requires the device under test (DUT) to be positioned at the geometric center of the anechoic chamber, which limits the flexible evaluation of modern wireless terminals. Although the spherical vector wave (SVW) method provides a rigorous electromagnetic mode expansion, its direct use in eccentric testing scenarios is prone to coefficient-domain overfitting. In the conventional coefficient-domain formulation, the increased involvement of high-order evanescent modes can lead to overfitting of physically insignificant coefficients, resulting in unstable and oscillatory reconstruction. To explain this behavior, an analytical periodicity model is developed and validated by numerical simulations, showing good agreement across all tested configurations. To overcome this limitation, this paper develops a unified 3D eccentric spatial–spectral composite operator for eccentric field synthesis by directly incorporating the three-dimensional offset into the field evaluation process. The proposed operator maps probe excitation weights to the translated 3D local test-zone field samples, thereby reformulating the synthesis problem from coefficient-domain fitting to field-domain matching. This field-domain formulation naturally downweights high-order modal components with negligible local-field contributions, thereby improving numerical stability. Numerical simulations in a 3D multi-probe anechoic chamber (MPAC) demonstrate that, under significant eccentric conditions, the conventional SVW method essentially fails, while the plane wave synthesis (PWS) method achieves less accurate reconstruction than the proposed scheme. In contrast, the proposed scheme maintains stable, oscillation-free reconstruction and consistently outperforms PWS by 5 to 15 dB across all evaluated scenarios. This work provides a promising solution for flexible 3D OTA evaluation of large-scale wireless terminals.

## 1. Introduction

With the large-scale commercialization of the fifth-generation (5G) communication systems and the rapid evolution of the sixth-generation (6G) technologies, advanced techniques such as massive multiple-input multiple-output (massive MIMO), millimeter-wave (mmWave), and smart beamforming have been widely deployed [1,2,3,4,5,6]. These technologies require tight integration between antennas and radio frequency (RF) front-ends (e.g., antenna-in-package, AiP), making traditional conductive testing infeasible due to the absence of physical RF interfaces [7,8,9]. As a result, over-the-air (OTA) testing has become a practical and widely adopted approach for evaluating the end-to-end radiated performance of modern wireless devices [10,11,12,13,14]. Among various OTA testing schemes, including reverberation chambers [15] and radiated two-stage methods [16], the multi-probe anechoic chamber (MPAC)-based approach has been established by standard bodies such as 3GPP as the core scheme for evaluating MIMO terminals and base stations, owing to its ability to reproduce complex 3D multipath fading channels in a controlled laboratory environment with high fidelity [17,18,19].

However, conventional MPAC testing imposes a strict geometric constraint: the DUT must be placed at the geometric center of the probe ring or spherical probe array, commonly referred to as the “center test zone” [19,20]. With the development of vehicle-to-everything (V2X) communications, large internet of things (IoT) devices, and massive MIMO base stations, the physical dimensions of DUTs have increased substantially [19,21,22,23,24,25]. Strictly enforcing center testing would require either extremely large anechoic chambers or a sharp increase in the number of probes and channel emulator (CE) resources, resulting in prohibitive hardware costs [26,27,28,29]. In addition, frequently repositioning large equipment to the geometric center of the chamber is both time-consuming and impractical. Mechanical centering remains valid when it is feasible, but for large DUTs, multiple antenna regions, or repeated test configurations, software-level eccentric synthesis can avoid repeated mechanical repositioning and associated repeatability uncertainties. There is therefore an urgent need for an OTA evaluation scheme capable of performing accurate 3D channel reconstruction directly in off-center regions of the chamber [23,30]. It is worth noting that such eccentric testing does not require expanding the test zone itself, as the test zone size is governed by the number of OTA probes and the required channel emulation accuracy rather than the physical location of the DUT.

Accurate field synthesis within an off-center test zone depends critically on the robustness of the channel emulation algorithm. The two mainstream approaches in MPAC systems are plane wave synthesis (PWS) and spherical vector wave (SVW) synthesis [31,32,33]. PWS is based on far-field and point-source assumptions and, while mathematically straightforward, its accuracy depends strongly on the geometric relationship between the target angle of arrival (AoA) and the probe positions. When the test zone is enlarged or displaced significantly from the chamber center, PWS cannot adequately account for near-field effects and wavefront curvature, leading to substantial reconstruction errors [24,25,34,35,36]. The SVW method, by contrast, is grounded in rigorous electromagnetic mode expansion theory and in principle provides accurate volumetric field description with rotational invariance, making it a more attractive candidate for 3D testing [37].

Despite this theoretical appeal, direct application of SVW in off-center scenarios encounters a numerical bottleneck. The mode truncation number *N* in conventional SVW expansion scales with the envelope radius *r* of the test zone (typically $N\approx kr+n_{1}$) [38,39]. In off-center configurations, the equivalent envelope radius typically increases substantially to cover the displaced DUT, thereby increasing the contribution of high-order evanescent modes. With a finite number of physical probes, this causes the conventional coefficient-domain formulation to overfit physically insignificant high-order coefficients, which in turn leads to unstable and oscillatory reconstruction. Ref. [40] proposed a MAPC scheme that can be applied to eccentric scenarios based on the traditional SVW. However, due to the expansion of the equivalent test zone, it requires thousands of probes, which significantly increases the hardware cost. A recently proposed enhanced SVW scheme [41] addressed this by solving directly in the electromagnetic field domain, so that high-order modal components are weighted according to their actual local-field contributions rather than fitted only as coefficient-domain targets. However, real-world wireless channels and large-device radiation patterns are inherently three-dimensional, and systematic investigation of this enhancement in 3D off-center configurations is still lacking.

It should be emphasized that the 3D off-center problem is not a straightforward extension of the previous 2D eccentric formulation. Previous MPAC studies have shown that the 2D channel assumption is not generally valid when elevation-domain characteristics are non-negligible, and that accurate OTA evaluation may require the reproduction of 3D radio channels rather than only azimuthal channel characteristics [20,42]. Recent reviews of MPAC OTA testing also distinguish 2D UE-oriented testing from 3D base-station or full-dimensional OTA testing, indicating that 3D OTA evaluation involves additional angular, spatial, polarization, and system-design requirements [17]. In a general 3D eccentric configuration, the offset vector can have arbitrary *x*-, *y*-, and *z*-components. After translation, each local test-zone sampling point undergoes coupled changes in spherical radius, polar angle, and azimuth angle with respect to the global probe-array center. Therefore, axial offsets, diagonal offsets, and elevation-dependent incident fields may lead to different eccentric-error behaviors that cannot be fully characterized by a 2D azimuth-only model.

To address this gap, this paper extends the enhanced SVW reconstruction method to 3D off-center testing scenarios, proposing a flexible 3D OTA evaluation scheme that directly synthesizes the target field within an off-center local test zone. The proposed method not only generalizes the enhanced algorithm from 2D to 3D anechoic chamber environments, but also fundamentally mitigates the coefficient-domain overfitting of high-order modes that afflicts conventional SVW under eccentric expansion. Extensive numerical simulations in a 3D MPAC show that the conventional SVW method largely fails under significant off-center conditions, and the conventional PWS method also shows clear accuracy limitations. The proposed scheme, by contrast, achieves stable and accurate reconstruction throughout. This work improves the practical applicability of SVW technology in complex OTA testing and offers a promising path toward 3D evaluation of large-scale wireless terminals with high flexibility.

## 2. Fundamentals of 3D Field Synthesis and Bottlenecks of Conventional Methods

### 2.1. Generalized Physical Model and Problem Formulation

A typical 3D MPAC system consists of *P* dual-polarized radiating probes distributed over a prescribed geometric topology (e.g., multi-ring spherical arrangement). Let the origin of the global coordinate system (GCS), denoted $O_{\mathrm{global}}$, coincide with the geometric center of the physical probe array. The position vector of the phase center of the *p*-th probe is $r_{p}$, with $∥r_{p}∥=R_{\mathrm{probe}}$. Each probe is driven by a complex excitation weight $w_{p}$, and the total synthesized electric field at an arbitrary spatial point r is

(1) $$E_{\mathrm{syn}}\left(r\right)=\sum_{p=1}^{P}w_{p}\;E_{p}\left(r\right),$$

where $E_{p}\left(r\right)$ is the complex radiated field of the *p*-th probe under unit excitation.

Let the center of the local test zone (TZ) be $O_{\mathrm{local}}$. In conventional center-testing, $O_{\mathrm{local}}\equiv O_{\mathrm{global}}$. In the off-center scenarios addressed in this paper, a deterministic 3D translation vector $r_{\mathrm{offset}}$ displaces the TZ from the chamber center. The field synthesis problem is then cast as an electromagnetic inverse problem: find the optimal excitation weight vector $w_{\mathrm{opt}}\in C^{P\times 1}$ such that $E_{\mathrm{syn}}$ best approximates the theoretical target field $E_{\mathrm{tar}}$ at *Q* discrete sampling points $\left\{r_{q}\right\}$ inside $V_{\mathrm{TZ}}$. Abstracting the spatial field mapping into a matrix operator Γ, the problem reduces to solving

(2) $$E_{\mathrm{tar}}=\Gamma \;w.$$

The core distinction among different synthesis algorithms lies in how the transfer operator Γ is physically and mathematically constructed.

### 2.2. Conventional Plane Wave Synthesis (PWS) Method

The PWS method is the most widely deployed field synthesis algorithm in MPAC systems. Based on the far-field approximation, the radiated field from each probe is modeled as an ideal uniform plane wave arriving at the TZ. The normalized field response of the *p*-th probe at point r within the TZ is therefore approximated as

(3) $$E_{p}^{\mathrm{PWS}}\left(r\right)\approx e_{p}\;e^{-jk{\hat{r}}_{p}\cdot r},$$

where ${\hat{r}}_{p}$ is the unit direction vector of the probe and $e_{p}$ is its polarization unit vector. In this framework, the generalized operator Γ reduces to the spatial phasing matrix $H\in C^{3Q\times P}$, assembled by stacking the plane-wave responses of all probes across all *Q* sampling points, and the synthesis equation becomes

(4) $$E_{\mathrm{tar}}=H\;w.$$

In practice, the probe weights w are determined via least-squares minimization.

The PWS method is computationally inexpensive and straightforward to implement. However, its accuracy is fundamentally bounded by the far-field geometric conditions of the centered quiet zone. When the TZ shifts by a large offset vector $r_{\mathrm{offset}}$, the wavefront curvature from each probe to the displaced TZ changes significantly, rendering the plane-wave assumption in H increasingly inaccurate. This mismatch leads to a progressive increase in synthesis error under off-center conditions, and a quantitative assessment of this degradation is provided in Section 4.

### 2.3. Traditional Spherical Vector Wave (SVW) Expansion

The SVW method is built on rigorous electromagnetic eigenmode expansion theory and is therefore capable of providing a volumetric, rotationally invariant field description. For a monochromatic target field $E_{\mathrm{tar}}\left(r\right)$ within the TZ, the SVW expansion reads

(5) $$E_{\mathrm{tar}}\left(r\right)=\frac{k}{\sqrt{\eta }}\sum_{s=1}^{2}\sum_{n=1}^{N}\sum_{m=-n}^{n}Q_{smn}^{\mathrm{tar}}\;F_{smn}^{\left(1\right)}\left(kr\right),$$

where $F_{smn}^{\left(1\right)}$ are standing-wave spherical vector wave functions of the first kind; s∈{1,2} indexes TE/TM polarization; *n* and *m* are the polar and azimuthal mode indices; *N* is the mode truncation number; and $Q_{smn}^{\mathrm{tar}}$ are the spherical wave spectrum (SWS) coefficients of the target field.

The radiated field from the *p*-th physical probe is mapped to the TZ center via the spherical wave addition theorem and Euler-angle rotation matrices, yielding the spectral-domain transfer coefficient $U_{smn}^{p}$:

(6) $$U_{smn}^{p}=\sum_{\sigma =1}^{2}\sum_{v=1}^{N^{\prime }}\sum_{\mu =-\min(v,n)}^{\min(v,n)}C_{s\mu n}^{\sigma v(3)}(k∥r_{p}∥)\;e^{j\mu \gamma_{z1}}\;d_{m\mu }^{n}\left(\gamma_{y2}\right)\;e^{jm\gamma_{z3}}{\left[\begin{array}{c} T_{\sigma \mu v}^{p,V}\\ T_{\sigma \mu v}^{p,H} \end{array}\right]}^{T},$$

where $C_{s\mu n}^{\sigma v(3)}\left(\cdot \right)$ are the third-kind spherical wave translation coefficients; $\left(\gamma_{z1},\gamma_{y2},\gamma_{z3}\right)$ are the Euler angles aligning each probe’s local coordinate system to the GCS; $d_{m\mu }^{n}\left(\gamma_{y2}\right)$ is the Wigner *d*-function; and $T_{\sigma \mu v}^{p,V/H}$ are the V/H polarization coefficients of the *p*-th probe. The complete recurrence relations of these functions can be found in [38].

In the SVW framework, Γ is realized through the spectral mode transfer matrix U. By cascading $U_{smn}^{p}$ for all probes and all modes, the system-level matrix $U\in C^{J\times P}$ (where J=2N(N+2) is the total number of modes) is assembled. The conventional SVW method then solves the spectral-domain matching equation

(7) $$Q_{\mathrm{tar}}=U\;w$$

to obtain the probe weights w.

The conventional SVW method achieves excellent accuracy in centered testing, where the required truncation order follows the classical rule $N\approx kR_{\mathrm{tz}}+n_{1}$ and remains small. When the TZ is displaced by $r_{\mathrm{offset}}$, however, the equivalent expansion radius must grow to encompass the offset DUT, forcing a large number of high-order modes into the system. With the finite probe count *P* constraining the available degrees of freedom, the conventional coefficient-domain formulation increasingly overfits physically insignificant high-order coefficients, ultimately causing severe reconstruction failure.

Although both the PWS and SVW methods perform well in traditional centered MPAC testing, each has an inherent limitation in eccentric scenarios. The PWS method faces a hard accuracy ceiling due to wavefront curvature mismatch, a consequence of the far-field assumption. The SVW method, while theoretically more rigorous, is susceptible to coefficient-domain overfitting: off-center translation forces a sharp increase in high-order truncation modes, causing the solver to overfit physically insignificant coefficients and thereby produce oscillatory and inaccurate field reconstruction.

## 3. Physical Mechanism of Eccentric Error and the Unified Spatial–Spectral Composite Operator

### 3.1. 3D Eccentric Topology Model and Target Field Modulation

In a 3D off-center testing scenario, the center of the local test zone $O_{\mathrm{local}}$ is displaced from the chamber’s global origin $O_{\mathrm{global}}$ by a deterministic 3D translation vector

(8) $$r_{\mathrm{offset}}=d\;\hat{e},$$

where $d=∥r_{\mathrm{offset}}∥$ is the eccentric distance and $\hat{e}$ is the unit direction vector of the offset. The position of the *q*-th sampling point in the global coordinate system is therefore

(9) $$r_{q}^{\prime }=r_{q}+r_{\mathrm{offset}},$$

where $r_{q}$ is its position in the local (TZ) coordinate system.

For a target plane wave characterized by the incident direction $\left(\theta_{l},\phi_{l}\right)$ and wave vector ${\hat{k}}_{l}$, the spatial translation introduces a phase modulation term Φ(d) into the target field evaluated at the displaced sampling points:

(10) $$\Phi \left(d\right)=e^{-jk\;{\hat{k}}_{l}\cdot r_{\mathrm{offset}}}=e^{-jkd\;\cos\;\psi },$$

where k=2π/λ is the free-space wavenumber and

(11) $$\cos\psi ={\hat{k}}_{l}\cdot \hat{e}$$

is the projection coefficient of the incident wave vector onto the offset direction. As *d* increases, Φ(d) traces a periodic trajectory on the complex unit circle, and it is this oscillatory modulation that acts as the physical origin of the coefficient-domain overfitting observed in conventional SVW methods under eccentricity.

Given the incident angles $\left(\theta_{l},\phi_{l}\right)$, the projection coefficients along the three Cartesian axes are

(12) $$\cos\psi_{X}=\sin\theta_{l}\cos\phi_{l},\;\cos\psi_{Y}=\sin\theta_{l}\sin\phi_{l},\;\cos\psi_{Z}=\cos\theta_{l}.$$

These expressions are used directly in Section 3.2 to obtain axis-specific period formulas. For a single plane-wave component, the translation-induced modulation factor reduces to $M\left(r_{\mathrm{offset}}\right)=\Phi \left(d\right)$.

### 3.2. Underlying Mechanism of Conventional SVW Divergence: Oscillation Periodicity Theory

The phase modulation term (10) is the fundamental oscillatory driver embedded in the target field as the offset distance *d* grows. In this section, we rigorously derive the spatial period of this oscillation and demonstrate its direct correspondence with the numerical oscillation divergence of the conventional SVW method.

We first derive the general oscillation period from the phase periodicity condition. As the offset distance increases by an increment Δd, the phase modulation term Φ(d) completes a full 2π rotation when Φ(d+Δd)=Φ(d). Substituting Equation (10) into this periodicity condition and solving for the minimum positive Δd yields the general oscillation period:

(13) $$\Delta d=\frac{\lambda }{\left|\cos\psi \right|}=\frac{\lambda }{|{\hat{k}}_{l}\cdot \hat{e}|}.$$

The geometric interpretation is immediate: the larger the projection of the incident wave vector onto the offset direction, the more rapid the phase variation and the shorter the oscillation period.

Substituting the Cartesian projection coefficients (12) into the general formula (13) gives the corresponding periods along the *x*-, *y*-, and *z*-axes:

(14) $$\Delta d_{X}=\frac{\lambda }{|\sin\theta_{l}\cos\phi_{l}|},\;\Delta d_{Y}=\frac{\lambda }{|\sin\theta_{l}\sin\phi_{l}|},\;\Delta d_{Z}=\frac{\lambda }{|\cos\theta_{l}|}.$$

The same periodicity argument can be extended to simultaneous equal displacements along the three Cartesian axes. For the most general 3D eccentric scenario where the TZ translates simultaneously along all three axes with equal displacement components (i.e., $\hat{e}=\frac{1}{\sqrt{3}}\left(\hat{x}+\hat{y}+\hat{z}\right)$), let the angular projection sum be defined as

(15) $$\alpha =\sin\theta_{l}\cos\phi_{l}+\sin\theta_{l}\sin\phi_{l}+\cos\theta_{l}.$$

The effective projection coefficient onto the diagonal direction is therefore $\alpha /\sqrt{3}$. Substituting this into Equation (13), the radial oscillation period measured along the 3D diagonal is

(16) $$\Delta r=\frac{\sqrt{3}\;\lambda }{\left|\alpha \right|},$$

where the factor $\sqrt{3}$ arises from the geometric conversion between the single-axis displacement *x* and the total radial distance $r=\sqrt{3}\;x$ along the space diagonal.

The specific numerical values predicted by Equations (13)–(16) for the three eccentric configurations studied in this paper are verified against simulation results in Section 4.2.

These closed-form expressions are valid as a first-order approximation based on the dominant propagating-wave components. In practice, spatial translation inevitably excites a large number of high-order evanescent modes (with mode index $n>kr_{\mathrm{tz}}$). The localized field perturbations introduced by these evanescent modes cause the measured oscillation periods in numerical simulations to deviate from the theoretical predictions by approximately 3% to 6%. This deviation is not a mathematical deficiency of the formulas; it is a direct physical manifestation of high-order evanescent mode interference. The accumulation of these evanescent modes is also the root cause of coefficient-domain overfitting in the conventional formulation, and this physical picture directly motivates the operator redesign in the following section.

### 3.3. Proposal and Construction of the Unified Eccentric Spatial–Spectral Composite Operator (Ψ)

The oscillation periodicity analysis in Section 3.2 reveals a fundamental obstacle: the growing phase modulation Φ(d) forces the conventional spectral-domain Equation (7) to demand increasingly high-order modes, which the finite probe array cannot support without overfitting the coefficient domain. To overcome this, we abandon the pure spectral-domain matching paradigm and instead propose a unified eccentric spatial–spectral composite operator Ψ, which transfers the field matching process to the physical spatial domain while algebraically incorporating the eccentric offset.

It is important to note that spatial eccentricity does not necessitate an expansion of the test zone radius $R_{\mathrm{tz}}$ [32]: the test zone size is governed by the number of OTA probes and the required channel emulation accuracy. The following derivation therefore keeps $R_{\mathrm{tz}}$ fixed.

Let *Q* discrete spatial sampling points ${\left\{r_{q}\right\}}_{q=1}^{Q}$ be uniformly distributed within the local off-center TZ $V_{\mathrm{TZ}}$. To make explicit how the offset enters the operator, we contrast $S_{\mathrm{offset}}$ with the standard centered sampling matrix $S_{0}$ used in [43]. In the centered case ($r_{\mathrm{offset}}=0$), the [3q−2:3q,j]-th block entry of $S_{0}$ evaluates the *j*-th spherical vector wave function at the local sampling coordinate $r_{q}$:

(17) $${\left[S_{0}\right]}_{q,j}=\frac{k}{\sqrt{\eta }}\;F_{smn}^{\left(1\right)}\left(kr_{q}\right).$$

In the eccentric case, the *q*-th sampling point is physically located at the globally translated coordinate $r_{q}^{\prime }=r_{q}+r_{\mathrm{offset}}$. The eccentric matrix $S_{\mathrm{offset}}$ is therefore obtained by substituting $r_{q}\mapsto r_{q}+r_{\mathrm{offset}}$ in every entry:

(18) $${\left[S_{\mathrm{offset}}\right]}_{q,j}=\frac{k}{\sqrt{\eta }}\;F_{smn}^{\left(1\right)}\;\left(k(r_{q}+r_{\mathrm{offset}})\right).$$

This substitution is the key modification with respect to $S_{0}$: the offset vector $r_{\mathrm{offset}}$ is directly absorbed into the argument of every wave function evaluation, thereby algebraically encoding the full 3D eccentric topology without introducing additional coordinate transformation operators. The convergence of the spherical wave expansion at the translated point $r_{q}+r_{\mathrm{offset}}$ is guaranteed provided that $∥r_{q}+r_{\mathrm{offset}}∥<R_{\mathrm{probe}}$, i.e., all sampling points remain within the source-free interior region bounded by the probe array, a condition satisfied by construction in the MPAC geometries considered in this paper.

By cascading $S_{\mathrm{offset}}$ with the spectral mode transfer matrix U introduced in Section 2.3, the unified operator is formally constructed as

(19) $$\Psi =S_{\mathrm{offset}}\;U,$$

where $\Psi \in C^{3Q\times P}$ provides a direct end-to-end mapping from the probe excitation weights w to the synthesized electric field at the eccentric sampling points. The construction of Ψ effectively replaces the overfitting-prone spectral matching step with a spatially grounded end-to-end formulation.

### 3.4. Field-Domain Matching Characteristic of Ψ and Robust Solution

The robustness of the proposed formulation should not be interpreted merely as a reduction of the raw algebraic condition number. Instead, the key difference lies in the optimization domain. Conventional SVW synthesis solves a coefficient-domain fitting problem, where all retained spherical-wave coefficients are treated as fitting targets. Intuitively, when the local test zone is displaced, the chamber-centred modal basis needs increasingly high-order modes to describe the shifted local field structure. When *N* becomes large, finite probe degrees of freedom may be spent on fitting high-order coefficients that contribute weakly to the actual field inside the local test zone, resulting in coefficient-domain overfitting and truncation sensitivity. In contrast, the proposed operator Ψ maps probe excitations directly to the translated field samples, so the least-squares objective is aligned with the field-domain FEVM metric. Consequently, high-order modal components with negligible field contributions are naturally downweighted through the field-domain projection, without introducing an externally designed modal taper.

To formulate the right-hand side of the synthesis equation, the target vector must include the phase modulation introduced by spatial translation. Let $E_{\mathrm{tar}}^{\left(0\right)}\left(r\right)$ denote the target field in the centered coordinate system. When the TZ is displaced by $r_{\mathrm{offset}}$, the modulated target field at the offset sampling points is

(20) $$E_{\mathrm{tar}}^{\mathrm{off}}\left(r\right)=E_{\mathrm{tar}}^{\left(0\right)}\left(r-r_{\mathrm{offset}}\right)\cdot M\left(r_{\mathrm{offset}}\right),$$

where $M(r_{\mathrm{offset}})$ is the complex phase modulation factor corresponding to the translation, consistent with the phase term Φ(d) derived in Section 3.1. Concatenating the modulated target field vectors at all *Q* sampling points yields the right-hand side vector $b\in C^{3Q\times 1}$.

Finally, the robust solution is obtained by combining the composite operator (19) with the modulated target vector b, which reduces the entire field synthesis problem to the end-to-end linear system

(21) b=Ψw.

The optimal probe excitation weights are obtained by minimizing the least-squares residual:

(22) $$w_{\mathrm{opt}}={\left(\Psi^{H}\Psi +\alpha I\right)}^{-1}\Psi^{H}b,$$

where ${\left(\cdot \right)}^{H}$ denotes the conjugate transpose, I is the identity matrix, and α≥0 is the Tikhonov regularization parameter. When α=0, Equation (22) reduces to the ordinary least-squares solution.

In the ideal numerical simulations in this work, α=0 or a very small value provides stable solutions because the least-squares objective is directly aligned with the field-domain FEVM metric. The improved robustness should therefore be interpreted as a consequence of field-domain objective alignment and implicit physical weighting, rather than as a universal reduction of the raw algebraic condition number. In practical MPAC systems, however, measurement noise, probe-response uncertainty, cable phase errors, and finite numerical precision may still require a small nonzero α. Therefore, α should be selected according to the noise level, calibration uncertainty, and required field-synthesis accuracy.

The above formulation also indicates how the method can be implemented in an MPAC system. From a practical implementation perspective, the proposed method can be mainly implemented as a software-level pre-compensation algorithm in the probe-weight calculation stage, without changing the physical MPAC topology such as the probe arrangement, chamber geometry, or channel-emulator architecture. Once the eccentric local test-zone position is known, the probe weights are calculated using the translated sampling positions and the calibrated complex probe-to-field responses. Therefore, the target field is synthesized directly in the eccentric local test zone rather than corrected by post-processing. However, practical deployment requires that the calibrated or accurately modeled probe-to-field responses be available over the eccentric local test-zone region. If the existing calibration only verifies the centered quiet zone, the calibration coverage should be extended to the eccentric region or supported by a validated probe-field model. Because Ψ is constructed from the estimated offset vector, residual offset errors introduce a mismatch between the assumed synthesis region and the actual LTZ and should therefore be included in the field-accuracy budget. Probe mutual coupling, chamber imperfections, cable phase errors, and calibration uncertainty can likewise be interpreted as residual errors in the calibrated probe-to-field operator; if they are accurately embedded in the calibrated responses, they can be included during weight synthesis, whereas unmodelled residuals will degrade the achieved FEVM.

The proposed formulation is not inherently restricted to far-field plane-wave assumptions, because it is constructed from SVW basis functions and calibrated probe-to-field responses. It can in principle be applied to compact radiating-near-field synthesis, provided that the local test zone is source-free, the translated sampling points remain inside the calibrated spatial region of the probe array, and the complex probe responses are accurately known. However, the method is not intended for arbitrary strongly reactive near-field measurement immediately adjacent to the DUT. The proposed synthesis operator also does not require the DUT radiation pattern or maximum directivity as an input, although highly directive DUTs may impose stricter requirements on field accuracy and calibration control in practical OTA evaluation.

Through the derivation in this section, we have established a complete and robust synthesis framework for 3D eccentric scenarios. The framework provides a mathematically consistent formulation and improves numerical robustness for reproducing complex spatial wavefronts at positions far from the chamber center. The following section validates these theoretical conclusions through comprehensive numerical simulation.

### 3.5. Complexity Comparison of PWS, Conventional SVW, and Proposed SVW

To clarify the numerical cost of the different synthesis strategies, let *Q* denote the number of spatial sampling points and let $P_{c}$ denote the number of independent probe excitation channels. For a dual-polarized array with $P_{\mathrm{ant}}$ physical probes, $P_{c}=2P_{\mathrm{ant}}$. The number of retained SVW modes is J=2N(N+2), which scales as $O(N^{2})$ with the truncation number *N*. The dominant construction, least-squares, and storage costs are summarized in Table 1.

For PWS, the operator H is obtained by evaluating ideal plane-wave responses at the sampling points, so both its construction and storage scale linearly with *Q* and $P_{c}$. For conventional SVW, the construction of U is dominated by the translation-coefficient calculation in Equation (6), where each modal coefficient involves the summations over σ, *v*, and μ. Since there are $O(N^{2})$ output modes and an $O(N^{2})$ internal modal summation for each excitation channel, the leading construction cost scales approximately as $O(P_{c}N^{4})$, while the memory required to store U scales as $O(JP_{c})$.

The proposed method shares the same U construction cost and additionally forms $S_{\mathrm{offset}}\in C^{3Q\times J}$ and $\Psi =S_{\mathrm{offset}}U$. These two extra steps require O(QJ) and $O(QJP_{c})$ operations, respectively. Assuming complex double-precision storage with 16 bytes per entry, explicit storage of U, $S_{\mathrm{offset}}$, and Ψ requires approximately $16(JP_{c}+3QJ+3QP_{c})$ bytes. For the simulation setting used in this work ($P_{\mathrm{ant}}=32$, $P_{c}=64$, and Q=2701), the matrix multiplication $S_{\mathrm{offset}}U$ at N=8 (J=160) requires approximately $24QJP_{c}=6.64\times 10^{8}$ real-valued floating-point operations. Representative runtime and memory results are further summarized in Table 2.

As shown in Table 2, when *N* increases from 4 to 12, the construction time of U increases from 7.147 s to 96.633 s, while the total construction time of Ψ increases from 7.685 s to 99.791 s. The additional field-domain construction therefore introduces only a moderate offline overhead under the investigated settings. Thus, constructing U remains the dominant cost as *N* increases, while the proposed method trades a modest increase in computation and memory for improved robustness under eccentric test-zone conditions.

### 3.6. Performance Metric: Field Error Vector Magnitude (FEVM)

To quantitatively evaluate field reconstruction accuracy, this paper adopts the field error vector magnitude (FEVM) metric, in the same spirit as evaluation criteria widely used in high-fidelity channel emulation literature. The metric measures the normalized discrepancy between the synthesized field and the theoretical target field over discrete sampling points in the local TZ. Since the electric field is vector-valued, the error is evaluated jointly over its three spatial components.

For the *q*-th sampling point at $r_{q}$, the point-wise FEVM (in dB) is defined as:

(23) $$\mathrm{FEVM}\left(r_{q}\right)=10\log_{10}\;\left(\frac{{∥E_{\mathrm{syn}}\left(r_{q}\right)-E_{\mathrm{tar}}\left(r_{q}\right)∥}_{2}^{2}}{{∥E_{\mathrm{tar}}\left(r_{q}\right)∥}_{2}^{2}}\right).$$

Here, ${∥\cdot ∥}_{2}$ denotes the Euclidean norm of the complex three-component electric-field vector. This point-wise definition is used in Section 4.4 to generate spatial error distribution maps over the spherical sampling surface of the eccentric TZ.

For quantitative comparisons in line plots and parameter sweeps, we employ the average FEVM over all *Q* sampling points. To avoid numerical instability at potential local field nulls, the average FEVM is defined as the ratio of total synthesized error energy to total target field energy across the TZ:

(24) $${\mathrm{FEVM}}_{\mathrm{avg}}=10\log_{10}\;\left(\frac{\sum_{q=1}^{Q}{∥E_{\mathrm{syn}}\left(r_{q}\right)-E_{\mathrm{tar}}\left(r_{q}\right)∥}_{2}^{2}}{\sum_{q=1}^{Q}{∥E_{\mathrm{tar}}\left(r_{q}\right)∥}_{2}^{2}}\right).$$

This definition provides a holistic scalar indicator of 3D volumetric field matching accuracy. Under this convention, a smaller (more negative) ${\mathrm{FEVM}}_{\mathrm{avg}}$ indicates lower reconstruction error and thus superior synthesis fidelity. FEVM should be interpreted as a field-level fidelity metric rather than a direct substitute for system-level OTA metrics such as channel correlation, receiver EVM, throughput, or MIMO capacity. A lower FEVM reduces amplitude, phase, polarization, and spatial-distribution errors in the synthesized test environment, but its exact impact on system-level metrics depends on the DUT antenna pattern, receiver architecture, SNR, channel model, and signal-processing chain.

## 4. Numerical Simulation and Analysis of Results

### 4.1. Simulation Parameters and Scenario Configuration

**Figure 1:**
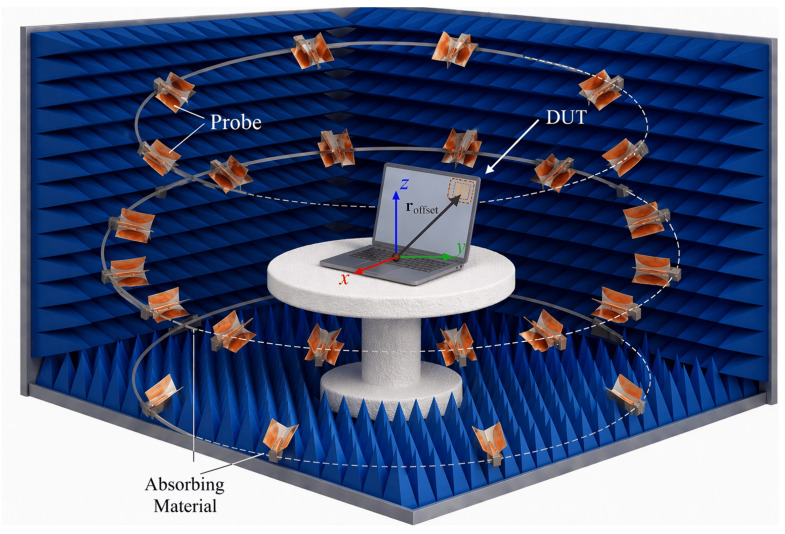
Topology of the probe array configuration in the 3D MPAC system.

To comprehensively verify the reconstruction performance of the proposed unified spatial–spectral composite operator Ψ, we constructed a typical 3D MPAC simulation testing environment. The probe array topology and device under test (DUT) configuration are illustrated in Figure 1. The coordinate system with the offset distance $R_{\mathrm{offset}}$ is also shown to highlight the eccentric setup.

The simulation system consists of P=32 dual-polarized Vivaldi antennas distributed on a truncated spherical surface with a radius of $R_{\mathrm{probe}}=3$ m. To achieve good spatial coverage and sampling uniformity in 3D space, the probe array is deployed across three horizontal rings at different elevation angles (θ). The upper ring is located at θ=60° and contains 8 probes with an azimuthal starting angle of ϕ=0° and an angular spacing of 45°. The middle ring lies in the equatorial plane at θ=90° and contains 16 probes with an azimuthal starting angle of ϕ=0° and an angular spacing of 22.5°. The lower ring is located at θ=120° and contains 8 probes with an azimuthal starting angle of ϕ=0° and an angular spacing of 45°.

The system operating frequency is set to $f_{c}=3.5$ GHz, and the radius of the local test zone (TZ) is configured as $R_{\mathrm{tz}}=0.75\lambda$. To evaluate the robustness of the proposed framework, we define representative target scenarios consisting of an ideal circularly polarized (CP) plane wave impinging from different spatial directions. Expressed in the spherical coordinate system, the field vector of the target incident wave is $E_{\mathrm{tar}}={\left[E_{r},E_{\theta },E_{\phi }\right]}^{T}={\left[0,1,-j\right]}^{T}$. Although a single CP plane wave is used as a canonical target field in the simulations, the proposed linear field-domain formulation is not limited to this case. More complex multipath channels with multiple simultaneous AoAs, angular spreads, and polarization mixtures can be represented by superposing the corresponding target field samples in the vector b. The achievable accuracy in such scenarios will depend on the spatial and polarization richness of the target channel and on whether the probe array provides sufficient degrees of freedom and calibrated probe-to-field responses over the LTZ.

To benchmark synthesis performance under both aligned and unaligned conditions, two specific incident paths on the equatorial plane are configured. Path A sets the angle of arrival to $\left[\theta_{\mathrm{inc}},\phi_{\mathrm{inc}}\right]=\left[90{}^{\circ},15{}^{\circ}\right]$. This direction does not align with any physical probe in the array, representing a general scenario that relies heavily on spatial wavefront interpolation. Path B sets the angle of arrival to $\left[\theta_{\mathrm{inc}},\phi_{\mathrm{inc}}\right]=\left[90{}^{\circ},0{}^{\circ}\right]$, which is strictly aligned with the phase center of a physical probe on the equatorial ring and thus serves as a baseline for evaluating the inherent synthesis accuracy.

These two paths serve as reference scenarios for comparing the three methods under typical incidence conditions. Building on these cases, the incident directions are varied in subsequent simulations to analyze the directional dependence of the algorithm and to validate the generality and robustness of the proposed method under diverse incidence conditions.

### 4.2. Numerical Validation of the Oscillation Period Theory

This section provides direct numerical verification of the analytical period formulas derived in Section 3.2. To resolve the oscillation structure clearly, the TZ center is swept along each offset direction in steps of 0.1λ over the range [0,4λ]. All three methods are evaluated simultaneously, with the spatial EVM defined in Equation (24) serving as the performance metric.

#### 4.2.1. Single-Axis X-Offset: Effect of Incident Azimuth on Oscillation Period

For a single-axis offset along $\hat{e}=\hat{x}$, the general period formula (13) predicts

(25) $$\Delta d_{X}=\frac{\lambda }{|\sin\theta_{l}\cos\phi_{l}|}.$$

Two incidence azimuths are examined to demonstrate the directional dependence of the period.

**Figure 2:**
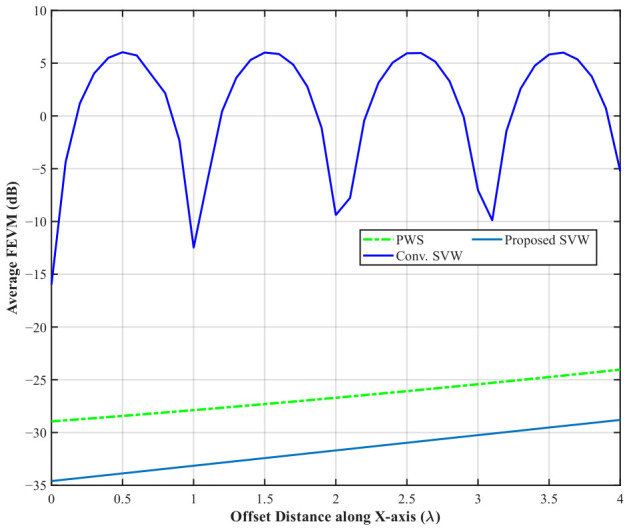
Average FEVM versus single-axis offset distance along *x* for Path ($\theta_{l}=90{}^{\circ}$, $\phi_{l}=11.25{}^{\circ}$).

Case 1 ($\theta_{l}=90{}^{\circ}$, $\phi_{l}=11.25{}^{\circ}$): substituting into (25) gives $\Delta d_{X}=\lambda /\left|\cos11.25{}^{\circ}\right|\approx 1.020\lambda$. Figure 2 shows the resulting EVM curves. The conventional SVW exhibits pronounced sawtooth oscillations that reach as high as +6 dB, significantly degrading the synthesized field quality over most of the offset range. The oscillation minima are located at d≈0,1,2,3,4λ, yielding a measured period of approximately 1.0λ, which is within 2% of the theoretical prediction and consistent with the first-order approximation analysis in Section 3.2. In contrast, the proposed SVW maintains a monotonically and smoothly increasing EVM throughout the entire range with no oscillatory behavior. It achieves a reconstruction accuracy consistently 5 to 6 dB superior to the PWS baseline, demonstrating that the composite operator Ψ has effectively eliminated the phase-modulation-induced oscillatory behavior predicted by the sawtooth theory.

**Figure 3:**
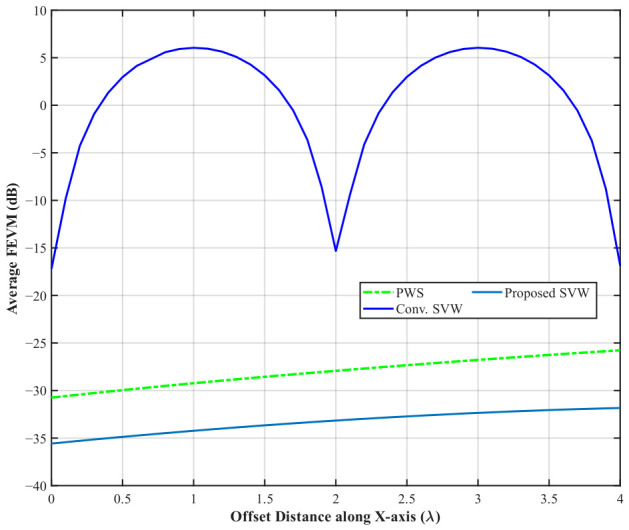
Average FEVM versus single-axis offset distance along *x* for Path ($\theta_{l}=90{}^{\circ}$, $\phi_{l}=60{}^{\circ}$).

Case 2 ($\theta_{l}=90{}^{\circ}$, $\phi_{l}=60{}^{\circ}$): the reduced projection coefficient $|\cos\psi_{X}|=|\sin90{}^{\circ}\cos60{}^{\circ}|=0.5$ yields a substantially longer theoretical period $\Delta d_{X}=\lambda /0.5=2.0\lambda$. Figure 3 presents the results. The conventional SVW oscillation period clearly doubles compared with Case 1, with minima located at d≈0 and d≈2λ, giving a measured period of ≈2.0λ in exact agreement with the prediction (error <1%). Comparing Figure 2 and Figure 3, the two-fold change in period when the incidence azimuth is rotated from 11.25° to 60° under the same offset direction directly validates the 1/|cosψ| dependence of Equation (13). The proposed SVW again demonstrates clean monotonic convergence and maintains its accuracy advantage over PWS across the full range, confirming that its suppression mechanism is independent of the incident angle.

#### 4.2.2. Three-Axis Comparison: Axis-Dependent Oscillation Behaviour

To verify the period formulas across all three Cartesian axes simultaneously, the TZ center is displaced along $\hat{x}$, $\hat{y}$, and $\hat{z}$ separately under the same incidence direction ($\theta_{l}=$ 90°, $\phi_{l}=$ 45°). The theoretical periods are:

(26) $$\Delta d_{X}=\frac{\lambda }{|\sin90{}^{\circ}\cos45{}^{\circ}|}\approx 1.414\lambda ,\;\Delta d_{Y}=\frac{\lambda }{|\sin90{}^{\circ}\sin45{}^{\circ}|}\approx 1.414\lambda ,\;\Delta d_{Z}=\frac{\lambda }{|\cos90{}^{\circ}|}\to \infty .$$

**Figure 4:**
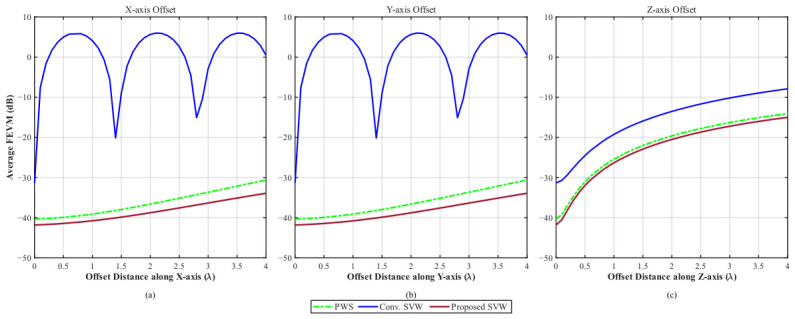
Average FEVM versus offset distance along the (**a**) *x*-, (**b**) *y*-, and (**c**) *z*-axes.

Figure 4 presents the three sub-plots. For the *x*- and *y*-axis offsets (Figure 4a,b), the conventional SVW exhibits nearly identical oscillation patterns with a period of ≈1.4λ, consistent with the equal projection coefficients along $\hat{x}$ and $\hat{y}$ at $\phi_{l}=$ 45°. The peak EVM reaches +6 dB in both cases, confirming the severity of the oscillatory reconstruction failure.

For the *z*-axis offset (Figure 4c), the behavior is qualitatively different: the conventional SVW no longer oscillates but increases monotonically with offset distance. This is a direct physical consequence of the formula. Since the equatorial incidence ($\theta_{l}=$ 90°) has zero projection onto the *z*-axis ($|\cos\psi_{Z}|=|\cos90{}^{\circ}|=0$), the phase modulation term Φ(d) vanishes for *z*-axis offsets, and the sawtooth mechanism is entirely absent. This result provides a particularly stringent test of Equation (13): the theory not only predicts when oscillations occur and at what period, but also correctly predicts when they do not occur at all.

Across all three axes, the proposed SVW maintains stable, monotonically increasing performance that consistently outperforms PWS, demonstrating that the robustness of the composite operator Ψ is independent of the offset direction.

Table 3 consolidates the theoretical predictions and simulation measurements for all four configurations examined in this section.

Across all configurations, theory and simulation agree within 2%, fully confirming the predictive validity of the sawtooth periodicity framework derived in Section 3.2.

### 4.3. Robustness to Mode Truncation Number *N*

A critical practical consideration in any SVW-based synthesis is the sensitivity to the mode truncation number *N*. In standard MPAC engineering, *N* is typically set conservatively to a value larger than $\left\lfloor kR_{\mathrm{tz}}\right\rfloor$. This section examines how this choice affects the three methods under both centred and eccentric conditions, and demonstrates a key operational advantage of the proposed method.

**Figure 5:**
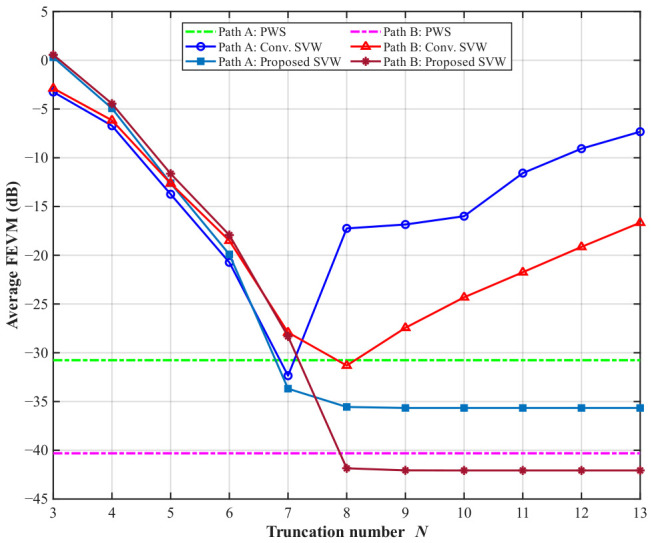
Average FEVM versus truncation number *N* at the centred position.

Figure 5 shows the average EVM as a function of *N* for the centred case ($r_{\mathrm{offset}}=0$) under both Path A ($\theta_{l}=90{}^{\circ}$, $\phi_{l}=15{}^{\circ}$) and Path B ($\theta_{l}=90{}^{\circ}$, $\phi_{l}=0{}^{\circ}$).

In the centred case, all three methods exhibit monotonic convergence as *N* increases, confirming that the baseline implementations are correct. Both the proposed SVW and the conventional SVW reach their optimal performance at approximately N=8, consistent with the theoretical estimate $N_{\mathrm{opt}}=\left\lfloor kR_{\mathrm{tz}}\right\rfloor +\mathrm{const}$. Even in this standard scenario, the proposed SVW already outperforms PWS by 5 to 10 dB across both paths, establishing a clear accuracy baseline before any eccentricity is introduced.

**Figure 6:**
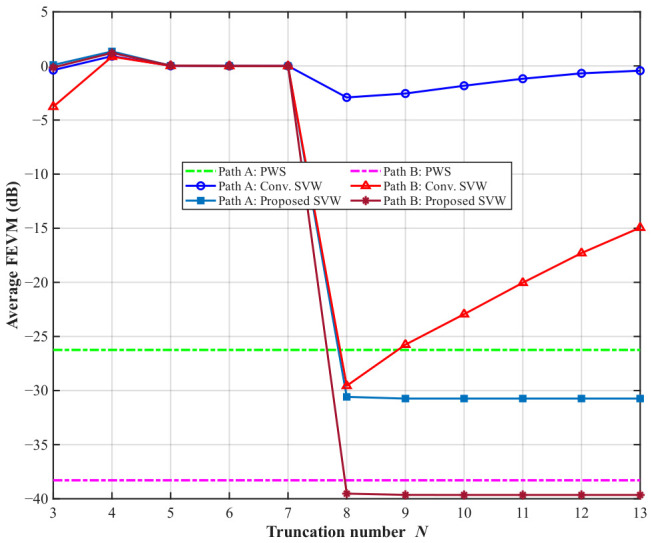
Average FEVM versus truncation number *N* at an off-centre position of 3λ.

Figure 6 presents the same comparison at an off-centre position of $∥r_{\mathrm{offset}}∥=3\lambda$ along the *x*-axis direction.

The off-centre case reveals a dramatic contrast. The conventional SVW achieves its best performance near N≈7 and then diverges as *N* is increased: Path A rises from ≈−2 dB back toward 0 dB, while Path B degrades more severely, reaching ≈−15 dB at N=13 after an initial drop to −30 dB. This asymmetric divergence between the two paths reflects the stronger 3D mode coupling under oblique incidence (Path B, $\theta_{l}=45{}^{\circ}$), which excites higher-order evanescent modes more aggressively and therefore drives the conventional solver into coefficient-domain overfitting at lower *N*. This behavior is a direct consequence of the mechanism analyzed in Section 3.2: increasing *N* beyond the critical value progressively introduces physically insignificant evanescent coefficients into the optimization, amplifying the oscillatory reconstruction error induced by the off-centre phase modulation.

The PWS method remains insensitive to *N* by construction and stays stable, but at a higher EVM floor (≈−26 dB for Path A, ≈−38 dB for Path B).

The proposed SVW, by contrast, preserves a clean L-shaped convergence profile for both paths even at 3λ offset. The EVM decreases sharply as *N* increases to 8, then plateaus at a stable floor of ≈−31 dB (Path A) and ≈−40 dB (Path B), surpassing PWS by 5 dB and 2 dB respectively. Increasing *N* beyond 8 adds only negligible noise rather than triggering renewed coefficient-domain overfitting. This robustness to *N* has direct engineering significance: the proposed algorithm can be deployed with a conservatively large *N* with reduced sensitivity to performance degradation, alleviating the need for the careful manual tuning that the conventional SVW requires.

In practical deployments, *N* can be selected according to the electrical size of the local test zone. A useful starting point is $N_{\mathrm{rec}}=\left\lceil kR_{\mathrm{TZ}}+\Delta N\right\rceil$, where k=2π/λ, $R_{\mathrm{TZ}}$ is the test-zone radius, and ΔN is a small safety margin of about 3–5 modes. In this work, $R_{\mathrm{TZ}}=0.75\lambda$, so $kR_{\mathrm{TZ}}\approx 4.71$, and N=8 provides a reasonable margin. For larger test zones or higher frequencies, *N* should be increased accordingly, but excessive values should be avoided because J=2N(N+2) increases the computational cost.

### 4.4. Reconstruction Fidelity Under Extreme Off-Centre Displacement

Having validated the periodicity theory and the truncation-number robustness, this section evaluates the three algorithms under extreme off-centre conditions to establish the practical limits of the proposed method.

#### 4.4.1. Long-Range FEVM Trend Along the 3D Diagonal

**Figure 7:**
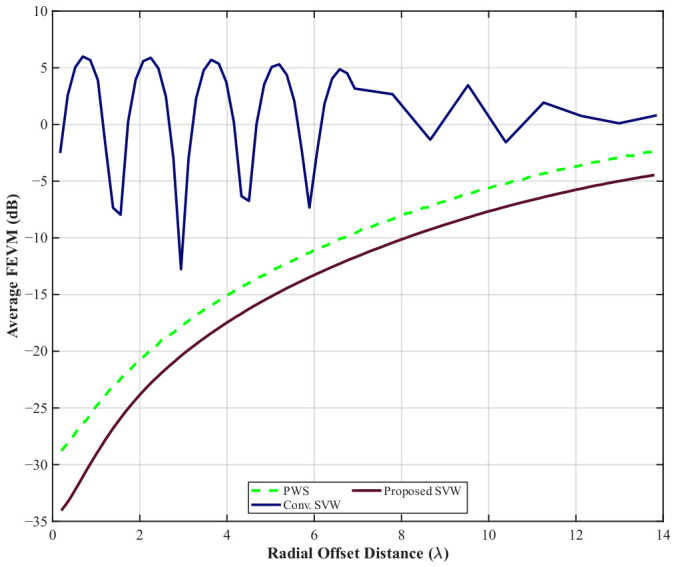
Average FEVM versus radial offset distance.

Figure 7 shows the average EVM over the full offset range [0,14λ] along the three-axis diagonal direction $\hat{e}=\left(1,1,1\right)/\sqrt{3}$ under Path ($\theta_{l}=90{}^{\circ}$, $\phi_{l}=11.25{}^{\circ}$). The maximum usable offset is not a fixed algorithmic constant; it depends on the probe-array radius, LTZ size, probe density, calibration coverage, target-field complexity, and the required FEVM threshold. A necessary geometric condition is that the entire eccentric LTZ remains inside the calibrated source-free region enclosed by the probe array, but the final usable range should still be verified using the application-specific field-accuracy criterion.

Three distinct regimes are observed. In the short-range regime (r<6λ), the conventional SVW undergoes violent sawtooth oscillations that repeatedly cross 0 dB, rendering the synthesis useless. The PWS EVM increases monotonically but steadily. The proposed SVW also increases monotonically but remains 10 to 15 dB below PWS throughout this regime, demonstrating a substantial accuracy advantage attributable to the field-domain projection in the composite operator Ψ.

Beyond approximately r=6λ, the amplitude of the conventional SVW oscillations gradually diminishes. This behavior is physically consistent with the asymptotic decay of the spherical Hankel functions: once kr≫N, the evanescent modes that drive coefficient-domain overfitting are naturally suppressed even within the conventional formulation, so the sawtooth fades and the conventional SVW EVM descends toward the PWS level. The proposed SVW continues to track smoothly, maintaining a consistent advantage over both methods. In the present configuration, if −10 dB average FEVM is used as a conservative field-accuracy criterion, the proposed method remains within the practical operating range up to a radial offset of approximately 8λ; larger offsets may still be acceptable for looser requirements but should be validated with the available calibrated probe-field coverage.

At very large offsets (r>10λ), the conventional SVW and PWS converge to comparable EVM values as both methods effectively reduce to a far-field approximation. This also explains why PWS can be close to the proposed SVW in some regimes: PWS is a stable low-order propagating-wave approximation, and its curvature error can remain moderate when the LTZ is electrically small relative to the effective probe-to-zone distance. The proposed SVW retains a modest but consistent advantage of approximately 1 to 3 dB, demonstrating the residual benefit of the composite operator Ψ even when the dominant sawtooth mechanism has subsided. These results confirm that the proposed algorithm provides reliable and superior reconstruction fidelity across the full practical range of off-centre displacements encountered in large-DUT MPAC testing. The method does not remove the physical need for sufficient probe density and spatial coverage; moderate probe reduction or layout non-uniformity may be tolerated, whereas overly sparse arrays will ultimately limit the available angular and polarization degrees of freedom.

#### 4.4.2. Spatial Error Distribution at Extreme Offset

**Figure 8:**
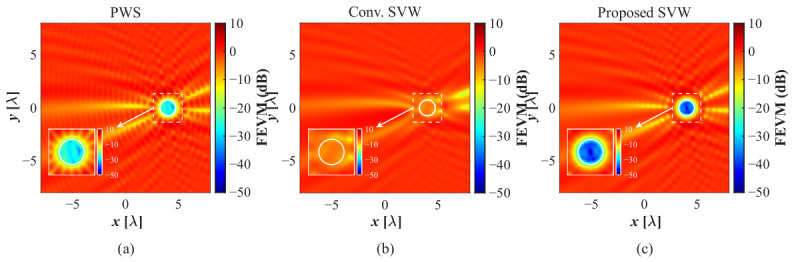
Point-wise FEVM distribution on the xOy plane at eccentric position x=4λ. The dashed rectangle and circle denote the 2.7λ×2.7λ observation window and LTZ boundary ($R_{\mathrm{tz}}=0.75\lambda$), respectively.

To visualize reconstruction quality across the spatial domain, Figure 8 presents the point-wise FEVM distribution over the xOy plane centred at the eccentric test zone position x=4λ along the *x*-axis direction, under the incident direction $\left(\theta_{l}=90{}^{\circ},\;\phi_{l}=11.25{}^{\circ}\right)$ with truncation number N=8. Each point (x,y) on the map represents the local FEVM value at that spatial location. The white dashed rectangle indicates the boundary of the enlarged observation region, corresponding to a 2.7λ×2.7λ window centred at the eccentric LTZ, and the white circle denotes the LTZ boundary with radius $R_{\mathrm{tz}}=0.75\lambda$. The inset in each panel magnifies the LTZ region to reveal fine spatial structure. The colour scale is identical across all three panels.

The PWS method (Figure 8a) produces a spatially structured interference pattern over the entire xOy plane. Outside the LTZ, the field error exhibits pronounced standing-wave-like fringes extending across the full field of view, a direct consequence of the far-field plane-wave approximation inherent to PWS, which becomes increasingly inaccurate as the test zone departs from the chamber centre. Within the LTZ, the inset reveals a relatively low-error core region, consistent with the average ${\mathrm{FEVM}}_{\mathrm{avg}}=-25.87\;\mathrm{dB}$ reported for this configuration.

The conventional SVW (Figure 8b) performs dramatically worse across the entire plane. The FEVM is elevated in large contiguous regions both inside and outside the LTZ, with values approaching or exceeding 0dB over much of the map. The inset confirms that even at the LTZ centre the error remains at a uniformly high level, yielding ${\mathrm{FEVM}}_{\mathrm{avg}}=-5.07\;\mathrm{dB}$ and constituting an effective synthesis failure. This degradation is a direct physical manifestation of the sawtooth oscillation mechanism analysed in Section 3.2: at x=4λ, the phase modulation term Φ(d) drives the strongest coefficient-domain overfitting in the high-order modes of the transfer matrix U, causing catastrophic reconstruction error throughout the LTZ.

The proposed SVW (Figure 8c) achieves a markedly superior spatial distribution. Within the LTZ, the inset reveals a deep-blue region with FEVM values predominantly below −30dB, yielding ${\mathrm{FEVM}}_{\mathrm{avg}}=-34.07\;\mathrm{dB}$—an improvement of 8.2dB over PWS and 29.0dB over the conventional SVW. Outside the LTZ, the proposed method also produces a smoother error pattern with lower peak values compared to both baselines, reflecting the spatial regularity enforced by the composite operator Ψ. The slight FEVM elevation at the LTZ periphery is physically expected: the reconstruction degrees of freedom provided by the 32-probe array are most efficiently utilised at the centre, and accuracy naturally decreases toward the boundary. This spatial uniformity within the LTZ is particularly important in OTA testing practice, where field quality criteria must be satisfied simultaneously at every sampling point within the quiet zone, not merely in an average sense.

## 5. Conclusions

This paper extends the enhanced spherical vector wave (SVW) reconstruction method to three-dimensional (3D) eccentric over-the-air (OTA) testing scenarios, proposing a flexible 3D OTA evaluation scheme based on a unified eccentric spatial–spectral composite operator Ψ. To explain the oscillatory behavior of the conventional SVW method under eccentricity, a sawtooth periodicity theory was rigorously derived and validated numerically, with prediction errors within 2% across all tested configurations. The composite operator Ψ is constructed by directly absorbing the 3D eccentric offset into the spherical vector wave function evaluations, thereby aligning the optimization objective with field-domain matching rather than coefficient-domain fitting. Numerical simulations in a 3D MPAC demonstrate that the proposed method maintains stable, oscillation-free reconstruction under significant eccentric conditions where the conventional SVW method essentially fails, consistently outperforming PWS by 5 to 15 dB. The proposed method also shows strong robustness to the mode truncation number *N*, removing the need for careful manual parameter tuning required by conventional SVW. This work offers a practically viable solution for the flexible 3D evaluation of large-scale wireless terminals in modern MPAC systems.

## Figures and Tables

**Table 1:** Numerical complexity and storage comparison of PWS, conventional SVW, and the proposed SVW method.

Method	Operator	Size	Construction Cost	LS Cost	Storage Cost
PWS	H	$3Q\times P_{c}$	$O(QP_{c})$	$O(QP_{c}^{2}+P_{c}^{3})$	$O(QP_{c})$
Conventional SVW	U	$J\times P_{c}$	$O(P_{c}N^{4})$	$O(JP_{c}^{2}+P_{c}^{3})$	$O(JP_{c})$
Proposed SVW	Ψ	$3Q\times P_{c}$	$O(P_{c}N^{4}+QJ+QJP_{c})$	$O(QP_{c}^{2}+P_{c}^{3})$	$O(QJ+JP_{c}+QP_{c})$

**Table 2:** Representative runtime and memory requirements for different truncation numbers.

*N*	*J*	Time of *U* (s)	Time of Ψ (s)	Working Memory (MB)
4	48	7.147	7.685	13.895
6	96	16.656	17.471	19.876
8	160	33.369	34.725	27.852
10	240	58.917	60.968	37.822
12	336	96.633	99.791	49.785

**Table 3:** Theoretical vs. measured EVM oscillation periods under all validated eccentric configurations.

Configuration	Offset Dir.	Theoretical Period	Measured Period	Error
$\theta_{l}=90{}^{\circ}$, $\phi_{l}=11.25{}^{\circ}$	$\hat{x}$	1.020λ	≈1.0λ	≈2%
$\theta_{l}=90{}^{\circ}$, $\phi_{l}=60{}^{\circ}$	$\hat{x}$	2.000λ	≈2.0λ	<1%
$\theta_{l}=90{}^{\circ}$, $\phi_{l}=45{}^{\circ}$	$\hat{x}$	1.414λ	≈1.4λ	≈1%
$\theta_{l}=90{}^{\circ}$, $\phi_{l}=45{}^{\circ}$	$\hat{y}$	1.414λ	≈1.4λ	≈1%
$\theta_{l}=90{}^{\circ}$, $\phi_{l}=45{}^{\circ}$	$\hat{z}$	*∞* (no oscillation)	confirmed	—

## Data Availability

The raw data supporting the conclusions of this article will be made available by the authors upon reasonable request to the corresponding author.

## Abbreviations

The following abbreviations are used in this manuscript:

OTA	Over-the-air
5G	Fifth-generation
6G	Sixth-generation
DUT	Device under test
EVM	Error vector magnitude
IoT	Internet of Things
MIMO	Multiple-input multiple-output
MPAC	Multi-probe anechoic chamber
PWS	Plane wave synthesis
RF	Radio frequency
SVW	Spherical vector wave
TZ	Test zone
V2X	Vehicle-to-everything
